# X‐Linked Hypophosphatemia Caused by a New Partial Insertion of LINE‐1 in the *PHEX* Gene

**DOI:** 10.1155/humu/3376327

**Published:** 2026-04-30

**Authors:** Dongmei Li, Wan Peng, Lu Kang, Jia Geng, Yu Lu, Jing Lu

**Affiliations:** ^1^ Department of Pediatric Nephrology, West China Second University Hospital, Sichuan University, Chengdu, China, scu.edu.cn; ^2^ Key Laboratory of Birth Defects and Related Diseases of Women and Children (Sichuan University), Ministry of Education, Chengdu, China, meb.gov.tr; ^3^ WCSUH-Tianfu, Sichuan Provincial Children′s Hospital, Meishan, China; ^4^ Institute of Rare Diseases, West China Hospital, Sichuan University, Chengdu, China, scu.edu.cn

**Keywords:** fibroblast growth factor 23 (FGF23), insertion, long interspersed element-1 (LINE-1), phosphate-regulating endopeptidase homolog X-linked gene (*PHEX*), X-linked hypophosphatemia (XLH)

## Abstract

X‐linked hypophosphatemia (XLH), primarily caused by mutations of the *PHEX* gene, is the most common cause of genetic rickets. Pediatric cases of XLH typically present with elevated levels of serum fibroblast growth factor 23 (FGF23), hypophosphatemia, rickets, and impaired growth. Here, we report a 2‐year‐old boy diagnosed with XLH, presenting with short stature, genu varum, and hypophosphatemia. Sanger sequencing revealed a novel mutation of the *PHEX* gene, comprising a 62‐bp poly‐T and a 421‐bp long interspersed element‐1 (LINE‐1) insertion into exon 22, which appears to induce the hypophosphatemia. This study presents the first documented case of XLH associated with a partial LINE‐1 insertion in *PHEX*. We suggest that LINE‐1 transposon element insertions be considered in XLH patients lacking other known mutations.

## 1. Introduction

X‐linked hypophosphatemia (XLH) is the most common cause of inherited rickets unresponsive to doses of vitamin D, with a prevalence of 1/20,000 [1, 2]. XLH is characterized by elevated circulating fibroblast growth factor 23 (FGF23), which affects renal phosphate reabsorption, leading to chronic renal phosphate wasting, hypophosphatemia, abnormal vitamin D metabolism, and impaired bone mineralization. Pediatric patients may typically present with symptoms such as rickets, short stature, lower limb deformities, and poor dental development. XLH is associated with variations, deletions, or duplications in the phosphate‐regulating endopeptidase homolog X‐linked gene (*PHEX*).

The *PHEX* gene is located in the Xp22.1 region, which comprises 22 exons and encodes a 749 amino acid protein, expressed mainly in osteoblasts, osteocytes, and odontoblasts. The protein has intracellular, transmembrane, and extracellular domains. As of the present, over 1000 distinct mutations associated with XLH have been recorded in the PHEX gene Locus Specific Database (LSDB) [3]. These mutations span the entire gene and include frameshift mutations, single nucleotide variants (SNV), copy number variants (CNV), and small or large insertions, deletions, and duplications. All are predicted to cause a loss of PHEX protein function. However, a partial LINE‐1 (L1) insertion in the *PHEX* gene has not been previously reported.

Over 40% of the human genome comprises parasitic elements known as interspersed repeat elements, categorized mainly as short interspersed nuclear elements (SINE), long interspersed nuclear elements (LINE), elements with long terminal repeats (LTR), and DNA transposons. LINEs are a family of autonomous retrotransposons, including L1, LINE‐2, and LINE‐3, making up a significant portion of the genome. L1 is the only active retrotransposable element in the human genome, accounting for approximately 17% of the human genome [4]. Although most L1 elements are nonfunctional due to mutations or deletions that inhibit retrotransposition, some remain active, generating new DNA copies through transposition processes and causing various genetic diseases by insertion, partial exon‐skipping, or chromosome inversions [5, 6].

Compared with adults, children experience rapid growth, making XLH′s effect on limb and trunk growth particularly pronounced. Timely diagnosis of XLH is crucial for improving final height outcomes and preventing additional endocrine complications. Treatment typically includes phosphate supplements and active vitamin D analogues. However, alternate diagnoses may lead to different treatment approaches, such as iron replacement in cases of autosomal dominant hypophosphatemic rickets [7]. Furthermore, burosumab, a humanized IgG1 monoclonal antibody targeting FGF23—which is consistently elevated in patients with XLH and plays a crucial role in its pathophysiology—has been shown to significantly ameliorate XLH symptoms.

In this study, we examined a Chinese boy diagnosed with XLH. Detailed genetic and immunological assessments revealed a partial L1 element insertion in exon 22 of the *PHEX* gene, contributing information to the LSDB and offering new insights into potential *PHEX* mutations.

## 2. Methods

### 2.1. Patient and Blood Sample Collections

A 2‐year‐old boy from a Chinese family was enrolled in this study. He presented with genu varum at 1 year and 6 months of age. Relevant clinical data, family history, and blood samples were collected during his first visit to our department at 2 years old. Peripheral blood samples were collected from the boy′s mother and matched healthy volunteers. Molecular analysis was conducted at the Genetic Diagnostic Laboratory of West China Hospital, and written informed consent was obtained from the child′s parents.

### 2.2. Genetic Testing and Analysis

#### 2.2.1. Whole‐Genome Sequencing and Analysis

Approximately 5 mL of peripheral blood was collected from the proband and his mother; DNA was isolated using the MagPure Tissue/Blood DNA LQ Kit (Magen Biotechnology Co. Ltd, D6312‐F‐96) by Thermo Scientific KingFisher Flex (Thermo Fisher Scientific Inc.). The DNA concentration was measured using the Qubit Fluorometer 3.0. Each sample was sequenced on the DNBSEQ‐T7 platform, generating at least 600 million reads in 2 × 150 paired‐end reads (30× coverage from ~120 Gb raw data for each sample). Human GRCh38 (hg38) was used as the reference genome.

Golden Helix VarSeq, the clinical genomics interpretation and reporting platform from Golden Helix Inc. (Bozeman, Montana, United States), was used for the analysis of genetic study results based on the VCF file. The variant annotation engine includes algorithms to identify the variant′s impact on genes using both public content RefSeq genes 109.20211119 (NCBI), ClinVar, Human Phenotype Ontology (HPO), Clinical Genome Resource (ClinGen), the Genome Aggregation Database (gnomAD), Online Mendelian Inheritance in Man (OMIM), the Human Gene Mutation Database (HGMD), and in silico predictors PROVEAN, MutationTaster, FATHMM, SIFT, and PolyPhen2. VarSeq was used to filter variants for quality (Read depth ≥ 6, Genotype Qualities ≥ 20 and Variant allele freq ≥ 1%), then POPMAX (maximum across populations) Alt allele Frequency ≤ 0.005 AND exclude variants in the noncoding region, OR “Pathogenic” and “Likely Pathogenic” in ClinVar, then exclude “Benign” and “Likely Benign” in ClinVar. Candidate genes associated with the phenotypes “Short stature” (HP:0004322), “Hypophosphatemic rickets” (HP:0004912), and “Rickets” (HP:0002748) were retrieved from the HPO database and cross‐matched with their corresponding disease‐related genes. The clinical manifestations of the proband and the inherited modes were considered to further exclude irrelevant genes.

### 2.3. PCR and Sanger Sequencing

Primers were designed for the *PHEX* gene variants using Primer 3 (https://primer3.org/). A pair of primers (Forward, 5 ^′^‐TTGGTATGGAACAGGCAGG‐3 ^′^ and Reverse, 5 ^′^‐AAGCAATGGGCGATGAAG‐3 ^′^) was used to amplify the *PHEX* intron 21 and exon 22. Long‐range PCR products, including *PHEX* intron 21 and exon 22, were obtained using the TaKaRa LA Taq (RR02MQ, Takara Biomedical Technology [Beijing] Co. Ltd.), according to the manufacturer′s recommendations. PCR conditions were as follows: 94°C, 1 min; 30 cycles of 98°C, 10 s; 68°C, 6 min; followed by 72°C, 10 min. PCR products were sequenced by Sangon Biotech (Chengdu, China). The reference sequence of *PHEX* used was GenBank NM_000444.6.

## 3. Results

### 3.1. Clinical Manifestations

**Figure 1:**
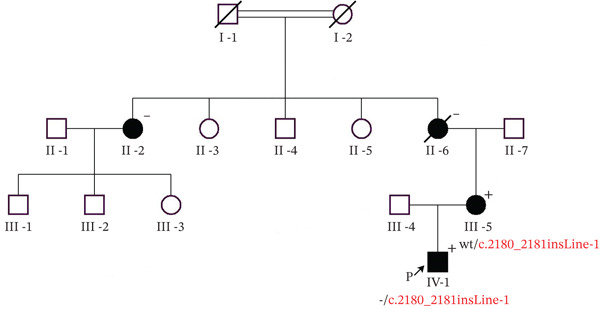
Family pedigree indicating a partial LINE‐1 insertion in *PHEX* gene. Genotypes of the proband and his mother are indicated. Black symbols indicate patients with hypophosphatemia phenotype. p: proband. Mutant variants are shown in red: c.2180_2181insLINE‐1. wt, wildtype.

The proband, a 2‐year‐old boy diagnosed with hypophosphatemic rickets, was enrolled at the Medical Genetics/Prenatal Diagnosis Centre of the West China Second University Hospital, Sichuan University. At the age of 1 year and 6 months, he presented with genu varum of the lower limbs, unresponsive to vitamin D and calcium supplementation. At 2 years and 1 month, he had a dental abscess involving the anterior teeth, followed by leg correction surgery. Despite treatment, he showed an unsteady gait and was prone to falling. Family history revealed that his grandmother′s parents were consanguineously married, and the grandmother and aunt had leg deformities. His mother, measuring 148 cm, also had O‐shaped legs in her childhood, which improved with correction. His grandmother is 120 cm tall (as shown in Figure 1).

Physical examination indicated short stature, a swaying gait, symmetrical thoracic cage, rib valgus, and O‐shaped legs. His height was 82.4 cm at 2 years old, below the average for children of the same age and sex (92 cm, −3 SD), and a sitting height/height ratio of 0.61.

Laboratory findings revealed decreased serum phosphate level (minimum 0.74 mmol/L, normal range 1.45–2.1 mmol/L), elevated serum alkaline phosphatase (ALP) (maximum 661 U/L, normal range 161.8–296.4 U/L), and elevated parathyroid hormone (PTH) (maximum 96.5 U/L, normal range 15–68.3 pg/mL), whereas serum calcium and 25 (OH) vitamin D levels remained normal (as shown in Table 1). No abnormalities were found in blood gas analysis, thyroid function, parathyroid ultrasound, or kidney ultrasound. X‐rays revealed O‐shaped knee joints bilaterally, with a slightly widened femoral and tibial epiphysis with irregular margins. The epiphysis of the ulnar shaft was also slightly widened, with reduced density.

**Table 1:** Laboratory data of the patient on admission.

	Before	After 6 months of conventional treatment	After 21 months of conventional treatment	After 9 months of somatropin therapy	Reference range
Height (cm)	82.4	88.5	92	100	
Weight (kg)	11.65	13	14	15.3	
Phosphate (mmol/L)	0.82	0.92	1.1	1.19	1.45–2.1
Calcium (mmol/L)	2.39	2.36	2.35	2.33	2.25–2.67
Alkaline phosphatase (U/L)	661	533	324	398	161.8–296.4
UN (*μ*mol/L)	14	18	21	16	17.3–54
25 (OH)D3 (ng/mL)	45.7	29.0	32.9	35.4	> 30
PTH (pg/mL)	96.5	85.90	55.4	35.2	15–68.3
TRP (%)	0.82	0.77	0.71	0.75	

Abbreviations: 25 (OH)D3, 25‐hydroxyvitamin D3; ALP, alkaline phosphatase; GFR, glomerular filtration rate; PTH, parathyroid hormone; TRP, tubular reabsorption of phosphate, which is calculated according to the formula of [1 − (urine phosphate × serum creatinine)/(serum phosphate × urine creatinine)] × 100*%*; UN, urea nitrogen.

Based on these findings, the diagnosis of hypophosphatemic rickets was confirmed. The proband was then treated with oral phosphate supplementation (60 mg/kg/day, six times daily) and calcitriol (50 ng/kg/day, twice daily). After 1.9 years of treatment, at 4 years and 3 months, his height remained below −3 SD, with a continued deviation from normal growth patterns; however, his lower limb deformities acquired significant improvement. Although his serum phosphate remained low, his ALP level normalized. Serum calcium and PTH levels maintained normal. Growth hormone therapy was introduced at age 4 for 9 months, resulting in a height increase of 6 cm over 6 months, but his height remained below −2 SD (Table 1).

### 3.2. Identification of a 62‐bp poly‐T and L1 Insertion in Exon 22 of the Proband′s *PHEX* Gene

**Figure 2:**
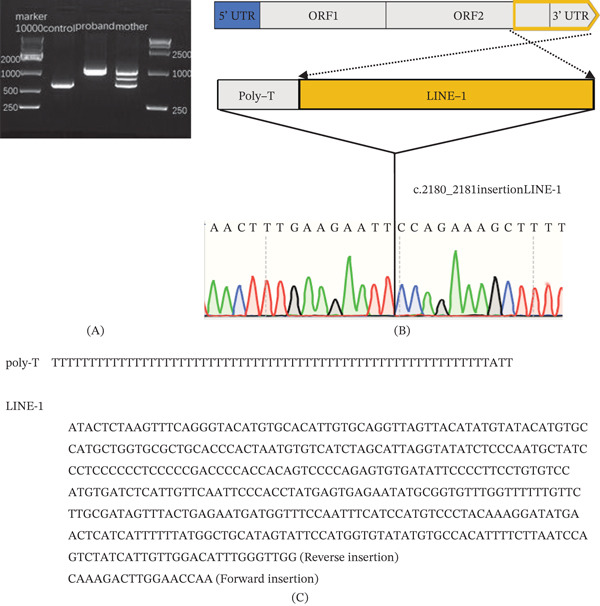
Partial LINE‐1 insertion in the PHEX gene. (A) Agarose gel electrophoresis showing long‐range PCR products. The longer PCR product (arrow) is present in the proband and his mother, but absent in the healthy control. (B) A 483‐bp insertion was identified in exon 22 of PHEX gene (c.2180_2181), consisting of a 62‐bp poly‐T duplicated sequence (grey) and a reversed partial LINE‐1 sequence (yellow). (C) Nucleotide sequence of the insertion showing the poly‐T region and the partial LINE‐1 sequence. Among these sequences, the initial 404 base pairs (bp) correspond to the reverse insertion of the LINE‐1 sequence, whereas the following 17 bp represent the forward insertion of the LINE‐1 sequence.

Since no SNV and CNV associated with vitamin D‐resistant rickets were found while the WGS BAM of the proband and his mother both showed that there was a breakpoint at chrX: 22,247,883 (GRCh38) in exon 22 of the *PHEX* gene, we designed a PCR primer targeting chrX:22247420‐22248034 (including part of intron 21, the whole exon 22, and part of the 3 ^′^‐untranslated region [UTR]). Sanger sequencing confirmed that the proband and his mother shared a common PCR band, which was not found in the healthy control sample (as shown in Figure 2A). Although the target region was chrX:22247420‐22248034, with an anticipated product size of 614 base pairs (bp), sequencing resulted in a fragment measuring 1013 bp. Of this fragment, only 530 bp aligned with the reference sequence, whereas the remaining 483 bp did not exhibit any alignment. Analysis of the mutant sequences revealed a 483‐bp insertion at chrX:22,247,883 within exon 22, which was also present in the proband′s mother, indicating her carrier status for the mutation. According to the ACMG/AMP variant criterion [8], the detected mutation can be considered pathogenic (PVS1, PM2, and PP4). Analysis of the source of the insertion showed that this mutation was composed of a 62‐bp poly‐T and a 421‐bp part of the L1 sequence (NCBI: MZ092700.1) (as shown in Figure 2B,C). The mother shows an unexpected band, which may result from heteroduplex formation during PCR.

## 4. Discussion

The diagnosis of XLH is based on family history, typical clinical manifestations, lower serum phosphate level, and genetic testing [9]. Actually, XLH is a genetic endocrine disease. Pathogenic mutations in the *PHEX* gene lead to reduced expression of its coding protein PHEX, which is located mainly in osteoblasts, osteocytes, and odontoblasts. The PHEX protein, a transmembrane protein composed of 749 amino acids, belongs to the M13 family of metalloendopeptidases and is thought to play a role in cell differentiation and phosphate sensing [10]. Although the mechanism by which PHEX deficiency elevated circulating FGF23 level remains unclear, elevated FGF23 is well‐established as a key contributor to the pathogenesis of XLH. FGF23 is mainly secreted by well‐differentiated osteoblasts and osteocytes, with the function of inhibiting phosphate reabsorption by kidney proximal tubules [11]. Additionally, FGF23 inhibits PTH secretion by parathyroid cells, and suppresses the expression of 1*α*‐hydroxylase in the principal tubular cells, leading to a decrease in the production of calcitriol. The resulting decrease in calcitriol further impairs sodium‐phosphate absorption in enterocytes. These mechanisms contribute to lifelong hypophosphatemia, leading to a range of bone disorders.

The boy in this report was diagnosed with XLH at age two, with the typical clinical manifestations of persistent hypophosphatemia and rickets that were unresponsive to standard vitamin D treatment. Although he received phosphate supplementation and calcitriol [12], his growth improvement was limited. Burosumab treatment was considered then [13]. However, it was declined by the child′s guardian due to the high cost, so growth hormone therapy was administered instead, resulting in a moderate improvement.

A new LSDB for PHEX, available on line (http://www.rarediseasegenes.com/), has now compiled almost all known PHEX variants, including variants from archived databases [14], hypophosphatemia genetic testing program [15], unpublished variants from clinical study data, and previously published literature. Mutations span the entire *PHEX* gene, including 22 exons, introns, and the 3 ^′^‐ and 5 ^′^‐UTR. As of October 18, 2024, the LSDB reports 1022 different variants, with SNV being the most common. Other mutations include splicing variants, small deletions, CNVs, small duplications, and insertions. Approximately 10% of clinically confirmed XLH patients may have undetected *PHEX* mutations [15]. Interesting, two Alu element (short‐interspersed element, SINE) insertions are recorded in this database, in the exon 16 and 22, respectively. This study is the first to report a partial L1 insertion in the PHEX gene.

Mobile elements, or transposons, make up nearly half of the human genome. Mobile element insertion (MEI) is believed to originate from ancient viral gene fragments that became embedded in the human genome as parasitic elements. Families such as Alu, L1, SINE‐VNTR‐Alu (SVA), and HERV‐K are commonly considered to be still active mobile elements [16, 17]. Transposable events can disrupt functional gene regions, impair gene function, alter transcript expression or splicing, and ultimately lead to diseases. Over 120 human genetic diseases have been reported to be associated with transposon‐mediated insertions, including hemophilia, Dandy–Walker syndrome, neurofibromatosis, and various cancer [6, 18]. However, in some cases, MEI may enhance target gene function [19].

Although the human genome contains more than 500,000 L1 sequences (an ancient genetic element constituting 17% of the human genome), a complete active L1 sequence is approximately 6 Kb in length [20], containing a 5 ^′^‐UTR promoter, two open reading frames (ORF1 and ORF2) [21], and a short 3 ^′^‐UTR. However, most L1 insertions are incomplete due to rearrangements, point mutations, and 5 ^′^‐truncation, rendering them inactive [22–24]. Only a small subset, around 80–100 L1 sequences, is considered still active in any given individual [25], with the set of active elements varying across individuals [26]. Here, we report a partial L1 insertion within a coding exon of the *PHEX* gene, causing typical XLH symptoms. However, L1 is not always deleterious to cells. Percharde et al. knocked down RNA transcription of L1 (to 80%–90% of normal levels) and found that the self‐renewal ability of embryonic stem cells was lost [27].

The genetic diagnosis process for this boy was quite complicated. Initially, WGS revealed breakpoints in exon 22 of the *PHEX* gene in the proband and their mother. Then Sanger sequencing also demonstrated the 483 bp insertion, consisting of poly‐A and truncated L1 elements. Following the ACMG guidelines [8], the c.2180_2181ins L1 variant is classified as pathogenic (PVS1, PM2, and PP4).

The identification of a partial L1 insertion in *PHEX* enriches the LSDB and offers new insights into *PHEX* variants. Nevertheless, certain limitations persist in our study. Firstly, we have not yet performed an in‐depth analysis of noncoding DNA, which may uncover previously unrecognized variations. Additionally, we were unable to obtain a blood sample from the proband′s grandmother, hindering our ability to investigate the intergenerational transmission of this insertion. Furthermore, due to the child′s significant clinical improvement, the parents have opted against further blood tests at this time. Consequently, we are temporarily unable to proceed with mRNA sequencing. We will remain cognizant of this recommendation and will endeavor to acquire the necessary samples for further verification should the opportunity present itself in the future.

## 5. Conclusion

In summary, we reported the first partial L1 insertion variant of the *PHEX* in a Chinese boy, with typical manifestations of XLH. When analyzing the sequencing results, if common variants such as point mutations were not found in a case with a high suspicion of XLH, it is important to think of the possibility of MEI, and then an in‐depth analysis of the sequencing results is required to avoid missing important findings.

## Author Contributions

Dongmei Li and Wan Peng contributed equally to this work and should be considered as co‐first authors.

## Funding

No funding was received for this manuscript.

## Conflicts of Interest

The authors declare no conflicts of interest.

## Data Availability

The data that support the findings of this study are available from the corresponding authors upon reasonable request.
